# A calming hug: Design and validation of a tactile aid to ease anxiety

**DOI:** 10.1371/journal.pone.0259838

**Published:** 2022-03-09

**Authors:** Alice C. Haynes, Annie Lywood, Emily M. Crowe, Jessica L. Fielding, Jonathan M. Rossiter, Christopher Kent

**Affiliations:** 1 Engineering Mathematics Department, University of Bristol, Bristol, United Kingdom; 2 Soft Robotics Group, Bristol Robotics Laboratory, Bristol, United Kingdom; 3 Bonnie Binary Ltd, Bristol, United Kingdom; 4 Faculty of Behavioural and Movement Sciences, Vrije Universiteit, Amsterdam, Netherlands; 5 School of Psychological Science, University of Bristol, Bristol, United Kingdom; University of Pennsylvania Perelman School of Medicine, UNITED STATES

## Abstract

Anxiety disorders affect approximately one third of people during their lifetimes and are the ninth leading cause of global disability. Current treatments focus on therapy and pharmacological interventions. However, therapy is costly and pharmacological interventions often have undesirable side-effects. Healthy people also regularly suffer periods of anxiety. Therefore, a non-pharmacological, intuitive, home intervention would be complementary to other treatments and beneficial for non-clinical groups. Existing at-home anxiety aids, such as guided meditations, typically employ visual and/or audio stimuli to guide the user into a calmer state. However, the tactile sense has the potential to be a more natural modality to target in an anxiety-calming device. The tactile domain is relatively under-explored, but we suggest that there are manifold physiological and affective qualities of touch that lend it to the task. In this study we demonstrate that haptic technology can offer an enjoyable, effective and widely accessible alternative for easing state anxiety. We describe a novel huggable haptic interface that pneumatically simulates slow breathing. We discuss the development of this interface through a focus group evaluating five prototypes with embedded behaviours (‘breathing’, ‘purring’, ‘heartbeat’ and ‘illumination’). Ratings indicated that the ‘breathing’ prototype was most pleasant to interact with and participants described this prototype as ‘calming’ and ‘soothing’, reminding them of a person breathing. This prototype was developed into an ergonomic huggable cushion containing a pneumatic chamber powered by an external pump allowing the cushion to ‘breathe’. A mixed-design experiment (n = 129) inducing anxiety through a group mathematics test found that the device was effective at reducing pre-test anxiety compared to a control (no intervention) condition and that this reduction in anxiety was indistinguishable from that of a guided meditation. Our findings highlight the efficacy of this interface, demonstrating that haptic technologies can be effective at easing anxiety. We suggest that the field should be explored in more depth to capture the nuances of different modalities in relation to specific situations and trait characteristics.

## 1 Introduction

Anxiety is a natural response of the body to perceived stress or danger. The sympathetic nervous system—the body’s “fight-or-flight” response—is activated by stressful stimuli, leading to physiological responses such as increased heart rate, altered heart rate variability and shallow, rapid breathing. If we experience this response too frequently, or the response is disproportionate to the stimulus, it can become an anxiety disorder, a chronic condition that impacts both our physical and mental health. Anxiety disorders are the most commonly reported mental health disorder worldwide and approximately one third of the population will be affected by an anxiety disorder during their lifetime [1]. It is also the sixth leading cause of non-fatal health loss globally and one of the leading causes of global disability [2]. For certain populations, anxiety symptoms appear to be particularly prevalent. In their meta review, Regehr et al. [3] highlight that approximately half of university students report moderate levels of stress-related mental health concerns, including anxiety and depression. They find that mindfulness-based interventions, among others, can significantly reduce symptoms of anxiety in this population.

The predominant treatment for anxiety orders is a combination of psychological therapies (typically Cognitive Behaviour Therapy, and applied relaxation therapy) and, when necessary, pharmacological treatment (e.g. selective serotonin reuptake inhibitors (SSRIs), and benzodiazepines). Therapy is costly, typically involving a minimum of 6–8 sessions, with an initial waiting time often of several weeks to months, and pharmaceuticals can be associated with significant side effects. Therefore, developing safe, effective and affordable non-pharmacological methods that reduce anxiety is an important area of research.

In this study we focus on test-anxiety in the undergraduate student population. During examination periods, students’ state anxiety increases significantly [4] and can become acute or cause longer term anxiety symptoms. This increase in state anxiety has been shown to alter heart rate variability [5], increase students’ blood pressure and heart rate [6] as well as increase cortisol levels in male students [4]. As well as being potentially detrimental to students’ general well-being and health, some studies have found state anxiety to have a negative impact on their academic performance [7, 8]. One study found that test-anxiety did not correlate with reduced examination scores but impacted the students’ sense of perceived imbalance between effort and reward [9] and that trait anxiety correlates with acute state anxiety before examinations.

Despite variation in the findings of studies investigating exam anxiety in students, they all highlight negative factors relating to increased state anxiety during the examination period. As researchers in affective haptics, we suggest that emotive haptic interfaces can be an excellent means of providing calming relief from anxiety symptoms. Touch is a powerful and intuitive sense. Non-noxious touch in the right context can lower physiological stress markers such as heart rate, blood pressure and cortisol levels, enhance heart rate variability, and increase levels of oxytocin, serotonin and dopamine associated with positive mood and well-being, reducing the sympathetic nervous activity associated with stress and anxiety [10–15]. As humans, we have functionally specialized nerve fibers (CT afferents) that respond to gentle, warm, caress-like touch with positive affective and physiological implications [16]. Aside from the physiological and emotional influence of touch, it is generally a widely accessible and intuitive channel, requiring no language skills or level of comprehension for its positive impact to be felt. It innately plays an important role in our well-being and healthy development from the moment we are born and even before [17]. It can also be a discreet and personal mode of interaction. These positive properties of touch lends it to being used as a modality for easing anxiety.

To this end, we describe the prototyping and realisation of a set of haptic interfaces that simulate calming touch interactions and investigate their potential to provide anxiety relief for students when experiencing anxiety about an examination. Through qualitative evaluation with students and design refinement, we develop a final artifact and conduct an empirical evaluation of this device to assess its efficacy as an aid for reducing anxiety.

## 2 Background

There are multiple products on the market that use touch-based methods for reducing anxiety. Examples include: Sensate, a vibration-based device that stimulates the Vagus nerve (a key component of the autonomic nervous system) [18]; Doppel, a wrist-worn device that produces a heartbeat-like vibration to induce states of calm or focus [19] and TouchPoints, wrist-worn devices that generate bilateral alternating stimulation via gentle vibrations on each side of the body, significantly reducing stress symptoms [20]. While these devices appear to be effective, they tend to use haptic stimuli as a mechanism to induce a desired goal state—such as reducing symptoms of nervousness prior to public speaking—and the devices are used for the achievement of that goal. That is, the user is consciously choosing to use the device for the purpose of relieving their anxiety symptoms.

There is an important difference between this kind of device and a device that not only has the targeted effect of reducing anxiety, but is engaging, comforting or calming to interact with in its own right and so would be an object that we intuitively turn to when feeling anxious. Examples of devices designed for the latter kind of interaction include personalised objects such as the HUG cushion-like wearable or Giggle Balls [21], designed to engage the user through comforting or playful interaction while enhancing well-being, or robotic animals such as Paro the seal [22] and Sefidgar et al.’s Haptic Creature [23] which are designed to engage the user by being soft, strokable and comforting while also reducing physiological stress markers.

We suggest that a device which is enjoyable and comforting to interact with in its own right, as well as providing a reduction in anxiety, will be more easily adopted and widely accessible. In addition, the anxiety-relieving effect of the device may also be enhanced when the affective and sensory experience of the device is perceived as congruent with the encalmed physiological state.

With this in mind, we looked to the natural touch experiences that have been shown to be calming—either subjectively or physiologically—and could be simulated in a device. In their review on touch interactions with humans, animals and robotic devices, Eckstein et al. [24] find that touch produces a calming and/or stress-relieving effect in the majority of interactions. However, this may be in part due to the lack of literature on negative touch interactions and due to the set-up of the studies presented. As discussed by Ellingsen et al. [25], the context and interpretation of touch plays a vital role in the emotional and physiological responses elicited. They discuss how responses to tactile stimuli are driven by competing “top-down” and “bottom-up” pathways. Top-down influences include conscious or unconscious pre-existing predictions, contextual interpretation and the effect of attention levels, mood and external cues on stimulus interpretation. Bottom-up influences include somatosensory processing and neural circuitry, the direct physical, physiological and neural processing of a touch stimulus. By taking inspiration from natural touch experiences and translating them onto a robotic interface we lose the social context of the original touch interaction and alter the top-down interpretation of the touch stimulus, running the risk of developing an interface that is perceived as creepy, awkward or uncomfortable to interact with. On the other hand, robotic interfaces can offer touch interactions that are controlled and predictable, and hence perceived as ‘safe’ compared to interacting with another human or animal. The system of affective touch is complex and personal and these factors need to be taken into consideration when designing haptic interfaces. With this in mind, the next section briefly explores the literature on calming touch interactions with animals, other people and the self to guide the design of our device.

### 2.1 Interactions with pets

Many people anecdotally find comfort and stress-relief from their pets. Petting a dog during a stress-inducing situation has been shown to significantly reduce anxiety levels [26, 27] and the presence of a familiar pet has been shown to be calming [28]. Interactions such as a person stroking their dog increases oxytocin levels, and to some extent decreases cortisol levels, in both owner and dog [29]. The purring frequency of cats aligns with the vibrations that are used in treatment for ‘bone growth/fractures, pain, edema, muscle growth/strain, joint flexibility, dyspnea, and wounds’ [30] so a purring cat on your lap can be not only soothing but also healing and strengthening. However, Eckstein et al. [24] review the literature on touch interactions with animals and find that the studies’ varied approaches and inclusion of confounding factors (such as verbal interaction) makes it difficult to draw clear conclusions on the direct influence of touch in animal interactions.

Robotic dogs and cats have been successfully trialed with people with dementia resulting in decreased stress and anxiety, reductions in the use of psychoactive drugs and pain medications, less agitated behavior and better quality of life [31, 32]. The aforementioned Paro [22] and Haptic Creature [23] have also demonstrated physiological effects associated with reduced sympathetic nervous system activity such as reduced blood pressure and breathing rate.

### 2.2 Interactions with people

Morrison [16] presents wide documentation demonstrating social touch as having stress-relieving and anxiety-reducing effects. They discuss the role social touch plays in physiological regulation as a “buffer” to stress in three contexts; thermoregulation such as snuggling, prosocial touch such as grooming and touch for proximity regulation to others. For students with high test anxiety, an examination is processed as a “threat” [33] and so the physiological regulation of social touch has beneficial implications. Many studies have shown the positive impact of warm, soft, slow caress-like touch on the skin which activates the CT afferents (nerve receptors that innervate the non-glabrous skin across the whole body) that are associated with pleasant touch and positive affect [34, 35]. When CT-afferents are stimulated, the sensation is typically perceived as pleasant and the properties of these nerve receptors has led to the “social touch hypothesis” [36]; that CT-afferents are attuned to close social interactions, resulting in positive affective and physiological responses to this kind of tactile stimuli. Hugging and being hugged has been shown in various studies to have a physiological impact, including reduced heart rates, increased oxytocin and pronounced parasympathetic activity [37, 38]. Studies find similar responses to general physical contact with a loved one such as holding hands [39]. Massage is another human touch interaction shown to relieve stress and anxiety [40]. People find comfort in feeling the calming presence of someone by their warmth or when feeling their slow breathing and heartbeat. This may be calming purely because of their presence and the association of being safe with them, but there could also be an element of physiological entrainment. Many of our physiological rhythms can entrain to external stimuli, for example, a baby’s heart rate is influenced by its mother’s when held [41, 42]. The breathing rate of sleeping babies entrains to the rocking rhythm of their cot [43] and the presence of a simulated heartbeat (such as the Mimo pillow [44]) or breathing (breathing bear [45]) soothes prenatal infants and improves their development. There is less evidence for breathing or heart rate entrainment in adults, but several haptic interfaces have successfully employed the simulation of a heart beat to regulate heart rate [46] and reduce anxiety [47] and synchronised breathing together can promote connection and relaxation [48].

It is important to note that the same touch stimuli could produce negative affective responses when presented in a different context. From studies with mediated touch interfaces that aim to calm, the findings are varied. For example, in different studies involving Hugvie [49–51], a huggable interface for phone conversations, the interface was shown to reduce state anxiety, cortisol levels and EEG power compared to the control cases, but these findings were not consistent across the studies. In addition, the physiological impacts such as reduced cortisol were not necessarily coherent with subjective measures.

### 2.3 Interactions with self

Self soothing touch-based interactions include physical practices such as yoga, which has been shown to reducing anxiety, and more so than metabolically matched walking [52]. This could be due to the movements in yoga stimulating the vagus nerve and balancing the autonomic nervous system activity [53]. Self-massage might also elicit these physiological effects [54]. Mindful breathing practices are another self-soothing method used for reducing levels of anxiety. Studies have shown that controlled slow, deep (sometimes referred to as diaphragmatic) breathing techniques increase parasympathetic dominance of the nervous system associated with “rest-and-digest” [55, 56]. They have also shown improved cardiovascular and cardiorespiratory function and respiratory efficiency as a result of deep breathing exercises [57–60]. Daily mindful breathing has been shown to significantly reduce test anxiety in students [61].

#### 2.3.1 Mindful breathing interfaces

There are many existing audio and/or visual mindful breathing guides such as meditation soundtracks and mindfulness apps [62]. Typically these require the use of a smartphone or tablet, which raises issues of inaccessibility as well as the avoidance of distracting elements of such devices. Many alternative interfaces and devices have been designed [63]. For example, Lotus [64] is an actuated origami flower with petals that open and close in response to your heart-rate to guide your breathing. ChillFish [65] and DEEP [66] are breath-controlled biofeedback games that uses gamification of biofeedback to help engage children in mindful breathing. However, few of them use a tactile modality beyond minimal vibrotactile cues via a smart watch or similar device.

Examples with a tactile element include Sato et al. [67], who developed a wearable vibrotactile display consisting of two transducers (audio speakers) on the upper and lower back that vibrate to create a phantom sensation travelling up and down the spine to guide the wearer’s breathing. Miri et al. [68] present a mixed design experiment (n = 97) with a personalised vibrotactile breathing pacer worn on the abdomen They find that the pacer reduces anxiety during a stressor task (compound remote associate word questions under time pressure). Paredes at al. [69] use a vibrotactile array embedded in a car seat to guide driver’s breathing which they find to be successful at reducing breathing rate during an in-lab experiment (n = 24). Bumatay et al. developed a meditation interface that delivered vibrotactile and/or audio breathing cues through a pillow that rested in participants’ laps [70]. The study details are limited, but they found the haptic-only modality to be the most subjectively relaxing and resulted in the largest decrease in stress based on a Stress Arousal Checklist. Further vibrotactile interfaces to encourage mindful breathing are Breathe with the Ocean [71], a blanket that mimics the tide coming in and out with vibration, and CalmMeNow [72] which generates vibration on the wrist and sternum through haptic bracelets, as discussed by McDaniel et al. [73]. The Breath Chair developed by Yanaka et al. [74] is a chair with embedded pneumatics in the back that simulate breathing. It was found to have no significant impact on participant’s reported anxiety or heart rate but did have a significant impact on skin temperature, suggesting that the breathing chair was calming while participants watched films that elicit fear or anxiety. Aslan et al. [75] present two somaesthetic designs that generate haptic sensations via an embedded servo-motor driven elastic deformable structure. One design is a soft toy that synchronises with your breathing as a meditation aid. Two expert meditators tried the device, suggesting that it would be useful for certain groups such as children to engage in mindfulness, although it could focus the child’s attention on the toy more than the body. Hall et al. [76] developed a haptic creature that is furry, warm, has an motor-driven off-centre mass to simulate purring, and a manually driven pneumatic chamber to simulate breathing. When looking at disturbing images, the haptic creature significantly reduced anxiety as measured via skin conductance, heart rate and forehead corrugator muscle changes. Ban et al. [77] developed Relaxushion. It is a cushion that simulates breathing via a motor-driven paddle that rotates two acrylic pieces in relation to one another creating the illusion of inflation and deflation. They posit that the cushion can control the rhythm of breathing for relaxation by overwriting somatic sensation, however they present no formal testing with the device. These studies show encouraging findings for the potential efficacy of haptic interfaces to ease anxiety. However, few of them conduct an empirical evaluation of the effect their device has on anxiety except for Miri et al. [68] who demonstrate the efficacy of conducting a large sample mixed design experiment for evaluating such interfaces. Other empirical evaluations include Yanaka et al. [74] and Hall et al. [76] who had mixed results from small sample sizes (n = 12 and n = 10 respectively), and Paredes et al. [69] who focus on use while driving.

## 3 Prototype development

To develop the prototype interface that we present and evaluate in this study we began with five prototypes inspired by the touch interactions discussed in the literature. These were evaluated in a focus group with 24 participants with the aim of identifying the prototype with the greatest potential for development into an effective anxiety aid. This section presents the core findings of this design process and the fabrication of the final interface that is at the heart of this study.

### 3.1 Primary prototypes

The preliminary prototypes were designed according to the following desired characteristics.

1. Incorporating a touch-based interaction to draw on the physiological benefits of touch and allow us to explore this underrepresented sense in the field of devices for anxiety relief.
2. Embodying an intuitive and inviting form, thereby being accessible without training or guidance.
3. Easing anxiety while offering an enjoyable, intuitive interaction.

With these characteristics as a guideline, and taking inspiration from the naturally calming interactions explored in the literature above, five prototypes were developed.

The prototypes were fabricated as circular cushions of similar size, weight and padding to ensure there was a consistent mode of interaction across devices, thereby reducing bias during focus group comparisons. Somatosensory behaviours of the prototypes included simulations of the natural sensations of breathing, purring and heartbeat to elicit the effect of calming interactions with pets, people and self. The fifth prototype employs a passive rather than active haptic interaction and is predominantly a visual interface. This was to give some comparison between the haptic modality and the more frequently used (in this field) visual modality. The passive haptic interaction in this prototype is that the intensity of the light can be altered by pressing into the cushion cover.

**Fig 1:**
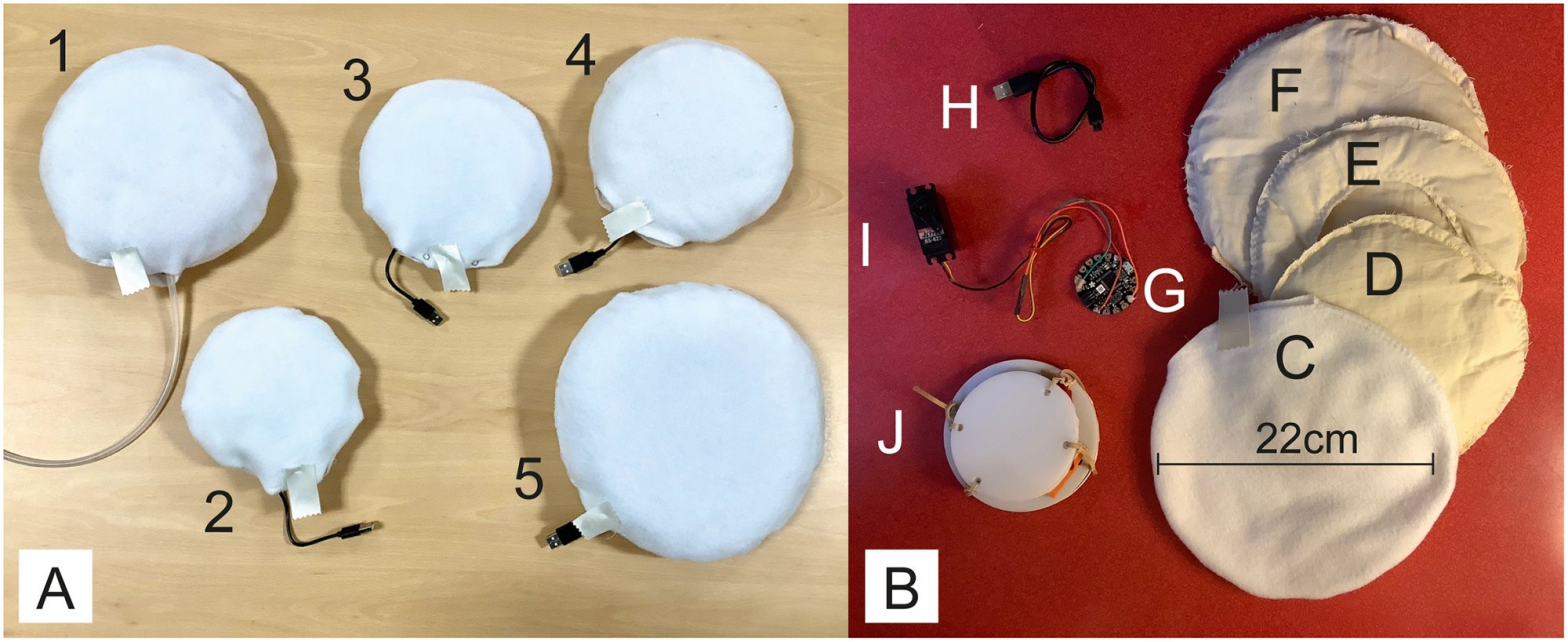
Photos of the primary prototypes. Panel A: Photos of the five primary prototypes presented to participants in the focus group. Panel B: Components of prototype number four (Purring and Breathing), to demonstrate internal components of the cushions; C) outer soft casing, D-F) inner padding, G) Adafruit Gemma circuit board, H) power cable, I) servo motor, J) acrylic disks to hold servo motor.

This resulted in the development of the five preliminary prototypes shown in Fig 1, described in Table 1 and demonstrated in action in the S1 Video. The mechanisms were embedded within each cushion and surrounded with padding to be undetectable except for their behaviour.

**Table 1:** Descriptions of the embedded mechanisms that drive the behaviours of the five prototypes shown in Fig 1.

No.	Behaviour	Mechanism
1	Breathing	Breathing simulated by the inflation and deflation of a silicone chamber tethered to an external manually-operated syringe pump. The chamber is sandwiched between two acrylic disks that are connected to one another with elastic straps to simulate the “ribs” that expand and contract within the cushion. The syringe pump is operated by hand at a rate of approximately 10–12 cycles per minute and 65 ml tidal volume.
2	Heartbeat	Heartbeat simulated by a DC motor driven off-centre mass protected by a hard plastic casing. A battery, Adafruit Gemma and motor driver inside the cushion power the motor which switches on in pulses just long enough for two revolutions, simulating a steady heartbeat rhythm. The heart rate is approximately 35 beats per minute, a very slow resting heart rate.
3	Purring	Purring simulated by a coin cell vibration motor, powered by an Adafruit Gemma and battery. The coin cell is fixed to a disk of cardboard between two layers of thick felt to both spread and dampen the vibration. It is activated sinusoidally, gentle increasing and decreasing in magnitude as though purring in sync with breathing at a rate of 17 cycles per minute, based on the sleeping and resting breathing rate of cats [78].
4	Purring and Breathing	Purring and breathing simulated by a servomotor held between two acrylic disks attached to one another with elastic straps. A plastic arm connected to the servo horn pushes the top acrylic disk up and down as the servo rotates clockwise and counter-clockwise. The vibration of the servomotor moving creates a purring sensation combined with the simulated rise and fall of breathing. The breathing rate is approximately 12 breaths per minute.
5	Light	A neopixel ring controlled by an Adafruit Gemma and battery is embedded into the cushion with one layer of padding on top and a layer of white polyester fabric to diffuse the light. The neopixel ring displays a slowly turning rainbow ring of light that rotates at approximately 10 cycles per minute.

These five prototypes were evaluated in a focus group to inform the development of the final artifact. Participants were recruited by email and word of mouth and gave written consent to take part in the study. This focus group was approved by the Faculty of Science Ethics Committee at the University of Bristol (study reference number: 100382). The participants were 24 students (18 aged 21–30, 6 aged 31–40; 7 female, 16 male, 1 preferred not to say gender). They were asked to feel and interact with the prototypes and answer questions via a worksheet. On the worksheet participants gave each cushion a rating based on the pleasantness of the interaction using a scale from 0 (really dislike the behaviour and would not choose to interact with it) to 10 (really like the behaviour and would actively choose to interact with it). They were asked to provide descriptions of the cushion behaviours and reasons for liking or disliking them. They then ranked the five prototypes from most to least pleasant.

**Fig 2:**
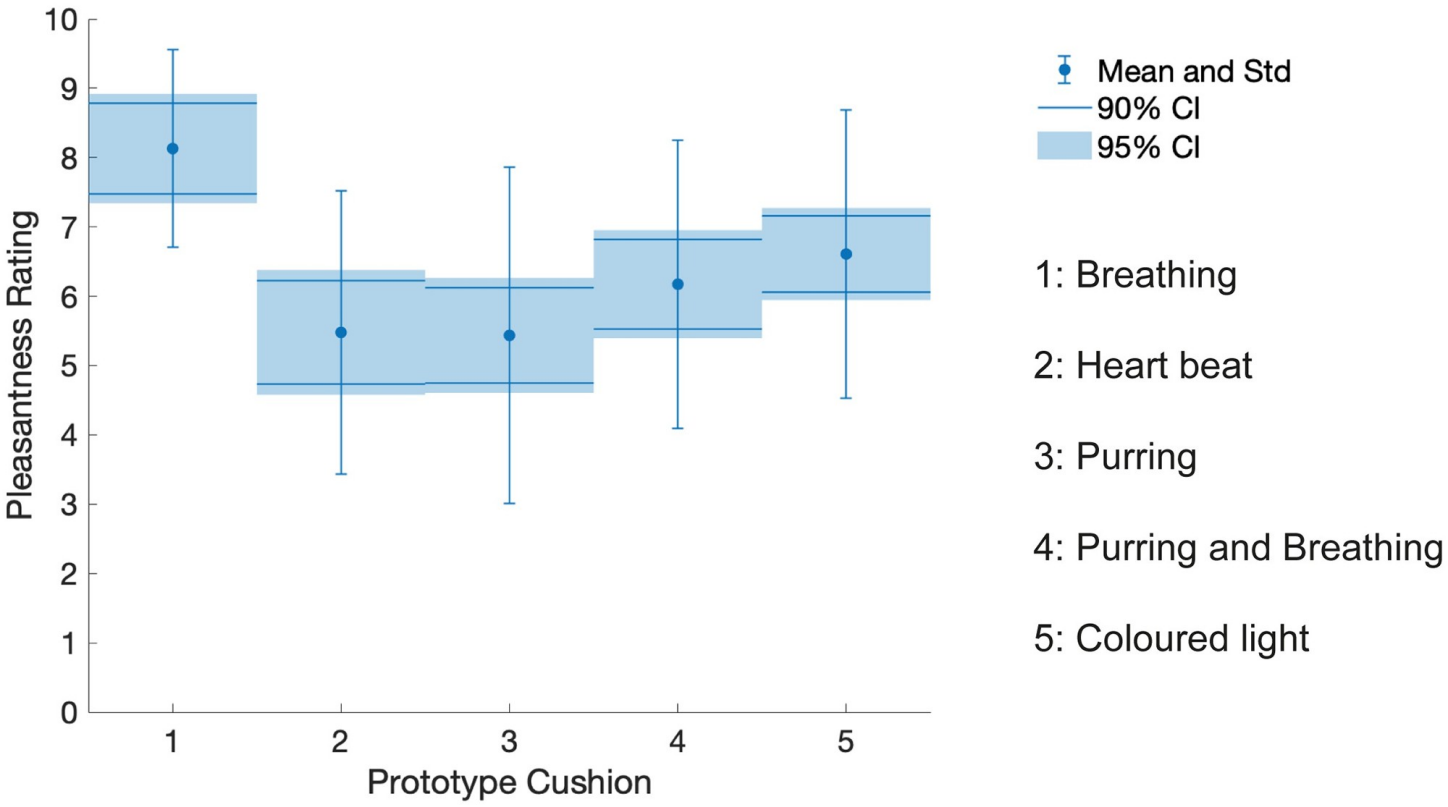
Participant ratings of the primary prototypes. Mean and standard deviation (std) of the valence ratings given by 23 of the 24 participants for each cushion. One participants’ data was excluded due to missing values. Confidence intervals of 90% and 95% are included for visual reference calculated using the Cousineau-Morey correction [79]. Participants recorded ratings as integer values between 0 (unpleasant) to 10 (pleasant).

As demonstrated by the pleasantness ratings shown in Fig 2, the breathing cushion was found to be the most pleasant with no participants giving it a negative rating. The ratings for the breathing cushion were significantly higher than the other prototypes (as shown by the 95% confidence intervals in Fig 2). This preference for the breathing cushion was reflected in the rankings participants gave as well as the comments and other answers (see S1 Fig). For example, 7 participants described the breathing cushion as ‘calming’, 3 described it as ‘soothing’ and 2 described it as ‘relaxing’. In addition, 9 participants commented that it ‘feels like breathing’ indicating that the simulation of breathing was effective and 3 associated it with the feeling of a cat in a positive context.

In this focus group and in prior work we conducted discussions with participants about their preferences (in the context of a calming cushion interface) for fabrics, colours and padding textures to guide the design of the final prototype. The feedback collected from these discussions fed into the final prototype design.

### 3.2 Final prototype

The focus group indicated that the breathing cushion generated the most pleasant sensations as well as being described as calming and soothing. Therefore, the breathing cushion concept was chosen to be develop into a final interface. The intention of this interface is to create a calming physical presence that eases anxiety. This could be due to multiple factors, for example, it could be due to the relaxing somatosensory feeling of hugging an external body that is slowly breathing (imparting a sense of safety and relaxation) or it could be by encouraging the holder to breathe slowly and deeply themselves either consciously or by physical entrainment of their breathing. Based on these potential factors, we made the assumption that modelling the breathing rate and style of the final artifact on the existing breathing methods that have been shown to reduce anxiety would enhance the effect of the device.

#### 3.2.1 Breathing rate, form and duration

Most therapeutic breathing methods involve deep or diaphragmatic breathing in which the diaphragm muscle contracts to inhale air deep into the lungs as discussed and demonstrated by Chen et al. [59]. Related studies typically refer only to the method of deep breathing, allowing participants to self-pace rather than prescribing a specific rhythm [80]. The typical breathing rate of an adult is between 12 and 18 breaths per minute [81] and Zaccaro et al. make a distinction of 10 breaths per minute as an inclusion criteria for slow breathing techniques [57]. There is a lack of evidence for the optimum ratio of inspiration duration to expiration duration for easing anxiety, but an equal inspiration and expiration rate was found to be the most effective according to various physiological markers in people new to yogic breathing [82]. Translating this to the device, we designed the cushion to fit comfortably on the belly and lower ribs when hugged so as to somatically encourage diaphragmatic breathing. The breathing rate was designed to be 10 breaths per minute with equal duration of inflation and deflation, with the intention of offering a slow and calming breathing sensation that is accessible for people to follow whether or not they have training in such breathing methods.

#### 3.2.2 Interface fabrication

**Fig 3:**
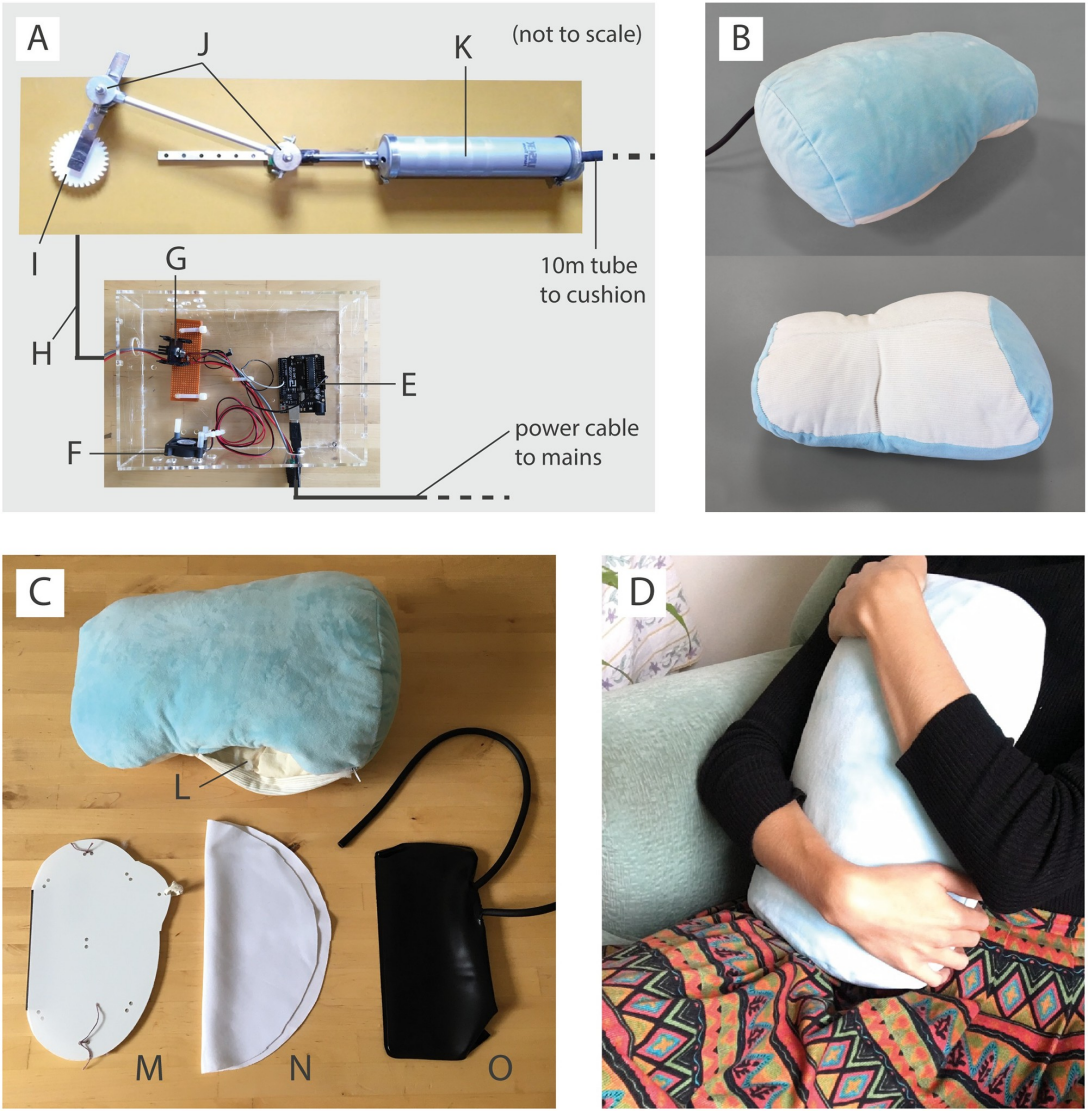
Diagram and photos of the final haptic interface. Panel A: Diagram of the mechanism driving the haptic sensation; E) Arduino, F) cooling fan, G) transistor, H) power cable to motor, I) motor (hidden) to drive crank, J) crank and slider, K) syringe pump that drives the inflation and deflation of the interface. Panel B: Photos of the interface front and back. Panel C: Inner parts of the interface; L) access zip, M) delrin casing for pneumatic chamber, N) smooth fabric to reduce audible friction, O) pneumatic chamber. Panel D: Photo of the interface being held in a home environment.

The textile interface of the cushion shown in Fig 3B–3D, was designed and fabricated to be ergonomically huggable and fit closely to the belly and chest when held. In doing so, the somatic sensation of breath provided by the interface is felt on the belly and lower chest, with the intention of bringing awareness to the depth of one’s breath and encourage the diaphragmatic breathing that has been shown to reduce anxiety. The cushion is 36 cm in length, 24 cm in width (at the widest span) and 15 cm thick. The main cover of the cushion was fabricated with extra soft washable polyester microfibre fabric to be enjoyable and comforting to touch, since soft fabrics like this were consistently preferred by participants. The inner curve of the cushion was fabricated with corduroy to add structure. The colours of light blue and white were chosen to be complimentary and calming, based on feedback from participants and the relevant literature [83]. The cushion was padded with polyester stuffing to protect and mask the inner mechanism as well as making the cushion pleasant to hug. Polyester was chosen as participants found it the most pleasant padding texture compared to other padding types such as grains, wool or memory foam. A concealed zip in one side provides access to the stuffing and mechanism inside.

#### 3.2.3 Interface mechanism

The interface is pneumatically driven by an external pump mechanism as shown in Fig 3A. An inflatable chamber, embedded within the centre of the cushion, consists of the bladder from a blood pressure monitor sandwiched between two 0.5 mm thick Delrin sheets lasercut to fit around the bladder and have smooth edges (Fig 3C). The sheets are fixed together with elastic straps around the bladder to hold it in place. They provide flexible support for the inflating bladder, holding it in place as well as amplifying the felt sensation. The bladder has a 30 cm long silicone flexible tube attached to it that comes out through a sewn hole in the bottom corner of the cushion. This tube is connected to a 10 m long flexible pipe extension linked to the pump mechanism, allowing for the mechanism to be kept out of sight and sound when being tested and to dampen vibrations from the pump and motor. The mechanism consists of a crank and slider powered by a windshield-wiper motor (Vauxhall Astra G MK4 1998–2009 front windscreen wiper motor) that drives a large syringe pump. The mechanism is shown running in the S1 Video. The mechanism pumps air in and out of the cushion chamber in a sinusoidal fashion and the crank was driven at a consistent speed to ensure equal duration of inflation and deflation. The crank was adjustable so that the optimum breathing rate and volume for the interface could be set at 500 cm^3^ tidal volume and 10 cycles per minute. This produces a minute ventilation of 5 Lmin^−1^, which lies within the typical human range for resting minute ventilation [84]. The actual tidal volume of the interface was measured to be 517 cm^3^. The breathing rate reduced slightly when the cushion was held and the time taken for 10 inflation-deflation ‘breath’ cycles was measured to be approximately 68 seconds.

When free to move, this breathing pattern on the cushion generates a change of 15 mm in cushion depth between minimum and maximum inflation. This is within the range of abdomen movement observed in humans while breathing, which Kaneko et al. found to be on average 6.8 mm for quiet breathing (around 15 bpm) and 32.9 mm for deep breathing (three full inhalations and exhalations) [85]. The stiffness of the cushion at minimum and maximum inflation was approximated using Hooke’s law by measuring the displacement of the cushion depth (perpendicular to the front face of the cushion) for a range of incident forces. This gave a stiffness of approximately 71 N*m*^−1^ for minimum inflation and 93 N*m*^−1^ for full inflation, a similar order of magnitude to the 50 N/m resting stiffness of the anterior abdomen wall in a healthy adult reported by Tran et al. [86].

## 4 Materials and methods

To evaluate the efficacy of the final interface at reducing anxiety we conducted an experiment in which we induced anxiety in our participants by using a social anxiety inducing situation (asking participants to perform difficult mathematics with peers watching). We compared the cushion with no-intervention control and guided meditation conditions. These conditions were tested in two identical intervention stages during the experiment; the first intervention was prior to the mathematics test to assess the impact of the cushion and meditation on anticipatory test anxiety, the second intervention was post mathematics test to assess the impact of the cushion and meditation on the induced test anxiety.

### 4.1 Experiment design

A mixed design was used to compare the efficacy of three interventions in reducing anxiety levels pre and post an anxiety-inducing situation. The dependent variable was anxiety levels, the within-subjects factor was time and the between-subjects factor was intervention. The within-subjects factor had four levels for each anxiety measure taken; baseline (T1), post intervention phase one (T2), post anxiety-inducing situation (T3) and post intervention phase two (T4). The three types of intervention, reflecting the between-subjects factor, were the interface, a mindful breathing meditation and a no-intervention control.

### 4.2 Participants

A total of 129 participants took part in this study, of which 96 were female and 33 were male (3 additional participants took part but their data was discounted because two of the participants filled out the questionnaire incorrectly and the third participant had clinically diagnosed anxiety). We conducted a sensitivity analysis, based on a desired power of 0.8, an alpha level of.05, and our 4 x 3 mixed design. With our sample of 129 participants, this allowed us to detect an interaction effect size as small as $\eta_{p}^{2}=.014$.

Participants were in the age range 18–36 years old (M = 20.49, SD = 2.75) and were a mix of undergraduate students from the University of Bristol who participated for course credit, as well as members of the general population recruited through word of mouth. All participants gave informed written consent prior to participation. Participants who had been clinically diagnosed with a mood or anxiety disorder were excluded from participation. This study was approved by the Faculty of Science Ethics Committee at the University of Bristol (study reference number: 96645).

### 4.3 Between-subjects factor: Interventions

The between-subjects factor was condition. Participants were assigned to one of three conditions:

#### 4.3.1 Control

Participants were asked to sit and do nothing for the control condition. They gave their mobile phones to the experimenter before the condition phase began to remove any potential distraction.

#### 4.3.2 Breathing meditation

The breathing exercise used was “Meditation Four: Breath and Body” adapted from Williams and Penman [87, 88]. The meditation is 8 m 18 s in duration and participants listened to it undisturbed via headphones.

#### 4.3.3 Cushion interface

Participants were asked to hug the cushion upright to their chest and belly as shown in Fig 3D. The pump and driver for the interface were hidden from view and the participants wore headphones to block any potentially distracting sounds.

Of the 129 participants, 44 participants were in the Control condition, 40 participants were in the Meditation condition and 45 participants were in the Cushion condition. In the few situations where a participant did not turn up for the experiment one of the team would act as a stand-in for the mathematics test to ensure that there were always three participants taking part in the test.

### 4.4 Within-subjects factor: Time point

The within-subjects factor was time point, marked by the four measurements taken during the experiment:

**T1**: Baseline measure at the beginning of the experiment. Pre intervention 1.
**T2**: Post intervention 1 and pre anxiety-inducing stimulus.
**T3**: Post anxiety-inducing stimulus and pre intervention 2.
**T4**: Post intervention 2 and final measure.

### 4.5 Anxiety-inducing stimulus

During the experiment, participants of all conditions were brought together for an anxiety-inducing event in the form of a verbal mathematics test. They were informed of the test after the baseline measures had been taken and prior to the first intervention. Anxiety therefore began to be induced between time T1 (baseline) and T2 (post intervention 1, anticipating test).

#### 4.5.1 Mathematics test

The test conformed with the Trier Social Stress Test paradigm [89] and was formatted in a timed PowerPoint that was presented to participants on a shared screen. Participants answered questions in front of each other to maximise the amount of social stress they felt and to heighten pre test anxiety.

### 4.6 Measures

The following measures were used to assess the participants during the experiment.

#### 4.6.1 State-Trait Anxiety Inventory (STAI)

**Fig 4:**
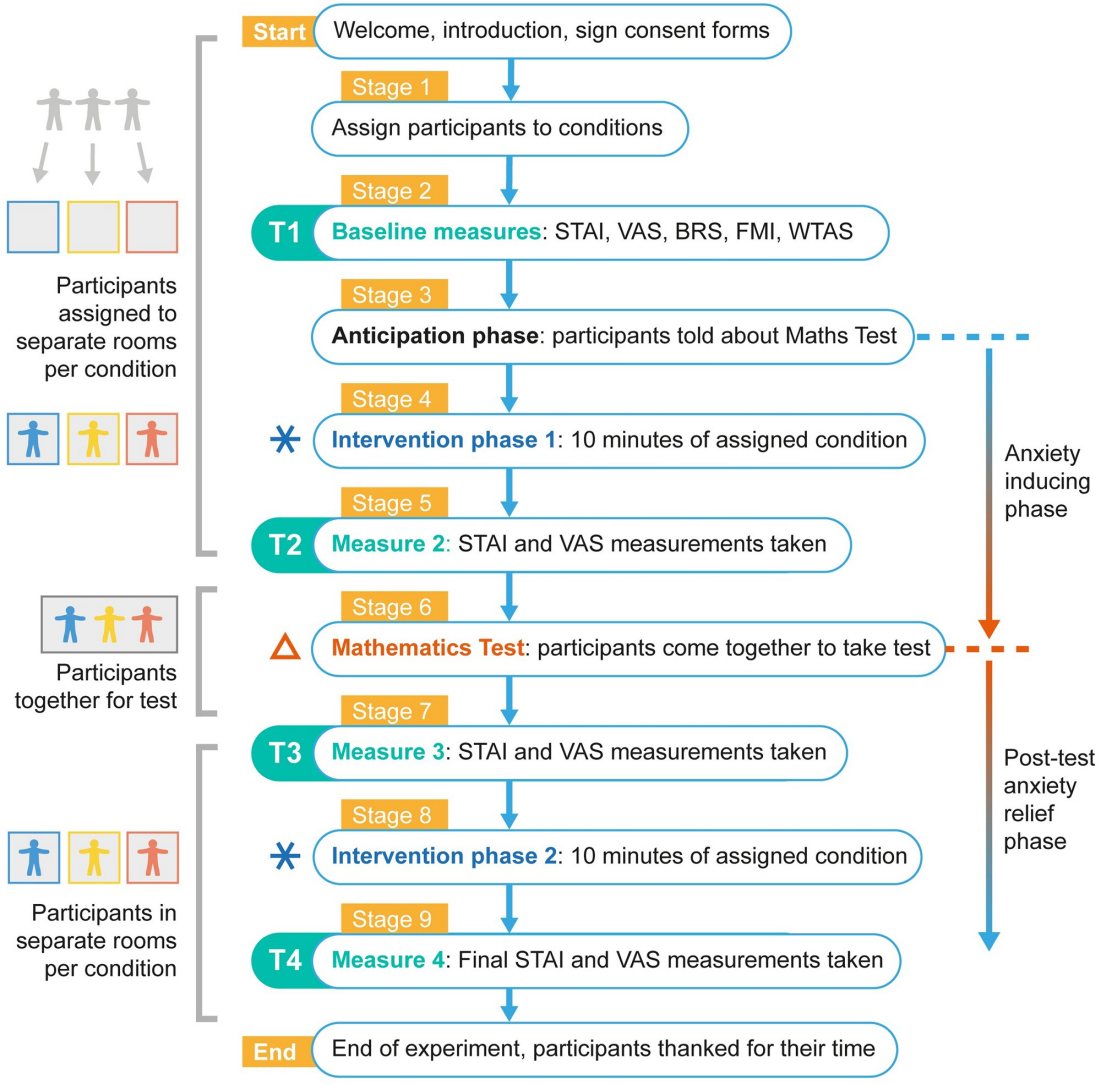
Stages of the experiment. Flow diagram illustrating the nine stages of the experiment. The four anxiety measures T1, T2, T3 and T4 are highlighted. The symbols * and △ label the intervention phases (*) and the anxiety-inducing mathematics test (△), as used in results figures later.

Anxiety levels were measured throughout the experiment at each time T1—T4 (as discussed later, see Fig 4) using the short-form Spielberger State-Trait Anxiety Inventory [90, 91] which presents 6 statements describing states or traits; “I feel calm”, “I am tense”, “I feel upset”, “I am relaxed”, “I feel content” and “I am worried”. Participants were asked to rate each statement according to how true it was for them using a 4-point scale of 1 (“not at all” for states or “almost never” for traits) to 4 (“very much so” for states and “almost always” for traits).

#### 4.6.2 Visual Analogue Scale (VAS)

Levels of anxiety and stress were measured using a visual scale from 1 to 100 on which participants drew a line to indicate their current state. Questions asked were“How anxious are you?” with scale Calm (1) to Anxious (100) and “How stressed are you?” with scale Relaxed (1) to Stressed (100).

#### 4.6.3 Brief Resilience Scale (BRS)

Trait Resilience was measured using the Brief Resilience Scale [92]. This measure was included for other purposes and is not analysed or discussed further here.

#### 4.6.4 Freiburg Mindfulness Inventory (FMI)

Trait Mindfulness was measured using the short-form Freiburg Mindfulness Inventory [93].

#### 4.6.5 Westside Test Anxiety Scale (WTAS)

Trait Test-Anxiety was measured using the Westside Test Anxiety Scale [94] to assess participants’ typical level of anxiety before an exam or test.

### 4.7 Method

The experiment consisted of nine stages as illustrated in Fig 4. Participants took part in groups of three (in situations where participants unexpectedly dropped out a member of the experimental team would act in their place) and were each pseudo-randomly assigned a condition; Control, Meditation, or Cushion. After they had been briefed and had signed a consent form, participants were assigned to a room according to their condition. When in their rooms they could not see or hear the other participants and had minimal distractions (no mobile phones, no windows, bare walls, headphones to block sounds). In their rooms, participants filled out a questionnaire providing demographic measures, baseline measures of trait variables and the first anxiety measure **T1**. The baseline measures were used to identify potential between-group differences and included measures of the trait variables Resilience (BRS), Mindfulness (FMI) and Test-anxiety (WTAS). The anxiety measures were STAI and VAS. All of the measures were carried out by participants on a computer, except for VAS for which they drew their response on paper.

After having had their baseline measures taken, the participants were then told that they will soon take a verbal mathematics test in front of the other participants. This anticipation phase is designed to heighten the participant’s anxiety levels. For the next 8 minutes 18 seconds (based on the duration of the meditation), the participants sat in their assigned condition (Intervention phase 1), either holding cushion, listening to the breathing meditation, or sitting quietly. Directly before going into the mathematics test another anxiety measure was taken, **T2**, using STAI and VAS. The participants then joined each other in one room for the verbal mathematics test, in which they took turns to answer the mathematics questions in front of each other. Directly after the test, participants returned to their rooms and the third anxiety measure, **T3**, was taken. They then sat in their respective conditions (Intervention phase 2) again for 8 minutes 18 seconds before completing the final anxiety measure **T4**. Having completed all of these stages, participants signed final consent forms and were thanked for their time.

## 5 Analysis plan

As a manipulation check we first conducted a one-way Analysis of Variance (ANOVA) to test between conditions at time T1 (baseline) to determine whether participants’ baseline measures were consistent across conditions. Next, we conducted a within-subjects t-test between T1 (baseline) and T3 (post test, pre intervention 2) to verify that the mathematics test was effective at eliciting anxiety.

To analyse the main experiment data, we first conducted a mixed 4 (time point: T1, T2, T3, and T4) by 3 (intervention: control, meditation, and cushion) ANOVA, with time as a within-subjects factor and intervention as a between-subjects factor.

As follow-up analyses we conducted t-tests whenever there was a significant main effect or interaction. We used a Bonferroni correction for multiple t-tests.

To investigate the effect of participants’ trait Test-Anxiety (WTAS) we conducted two analyses. First, we conducted a mixed measures ANCOVA with WTAS as the covariate measure to verify any significant interaction. Second, we focused on the anticipation phase between T1 (baseline) and T2 (post intervention 1, anticipating test). We used a Pearson product-moment correlation to determine the relationship between participants’ trait Test-Anxiety (WTAS) and their change in STAI Anxiety between T1 (baseline) and T2 (post intervention 1, anticipating test). We expected there to be a positive correlation for the Control case, i.e. participants with greater trait Test-anxiety would experience a greater increase in anxiety when anticipating the mathematics test, but we were investigating whether the intervention may have altered this for participants in the other two conditions.

## 6 Results

A one-way ANOVA for each of the baseline measures confirmed that there was no significant difference between groups at time T1 (baseline); STAI anxiety (F(2, 126) = .239, p = .788, $\eta_{p}^{2}=.004$), Resilience (F(2, 126) = 1.02, p = .364, $\eta_{p}^{2}=.016$), Mindfulness (F(2, 126) = 1.08, p = .341, $\eta_{p}^{2}=.017$) and Test-anxiety (F(2, 126) = .124, p = .884, $\eta_{p}^{2}=.002$). A within-subjects t-test indicated that anxiety as measured by the STAI was significantly higher at time T3 (post test, pre intervention 2) than at time T1 (baseline), verifying that the mathematics test was effective at inducing state anxiety; M difference = 5.71, SD difference = 12.2, t(128) = 5.295, p <.001, d = .47.

**Fig 5:**
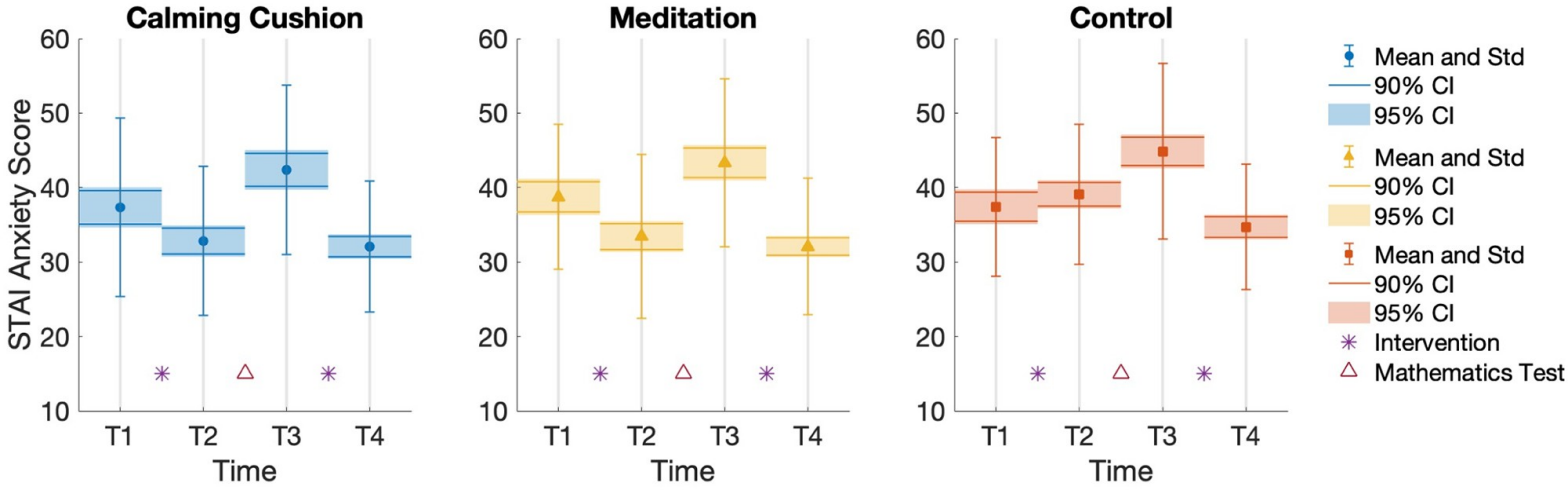
STAI anxiety scores for within-subjects factor. Comparison of within-subjects factor (time) for mean STAI Anxiety measures at each time T1 (baseline), T2 (post intervention 1, anticipating test), T3 (post test, pre intervention 2) and T4 (post intervention 2), including standard deviation (Std) errorbars, as well as the 90% and 95% confidence intervals (CI). Confidence intervals are calculated using the Cousineau-Morey correction [79] and therefore provide visual reference as to statistically significant changes over the within-subjects factor of time.

The STAI was our main anxiety dependent variable, the results of which are shown in Fig 5. A mixed 4 x 3 ANOVA on anxiety (with a Huynh-Feldt correction due to a violation of sphericity) showed a main effect of time on anxiety: (F(3, 378) = 53.74, p <.001, $\eta_{p}^{2}=.299$); No main effect of condition (F(2, 126) = 1.44, p = .240); and a significant interaction between time and condition (F(2, 126) = 2.19, p = .05, $\eta_{p}^{2}=.034$).

A between-subjects t-test indicated that, at time T2 (post intervention 1, anticipating test), STAI responses in the Cushion condition (M = 32.8, SD = 10.0) were significantly lower than STAI responses in the Control condition (M = 39.1, SD = 9.36); t(86.8) = 3.05, p = .003, d = .65. STAI responses in the Meditation condition (M = 33.4, SD = 11.0) were also significantly lower than in the Control condition; t(76.96) = 2.53, p = .013, d = .56. STAI responses in the Cushion and Meditation conditions were statistically indistinguishable; t(79.4) = 2.62, p = .793, d = .057. VAS anxiety scores and VAS stress scores showed a similar pattern: dropping between T1 (baseline) and T2 (post intervention 1, anticipating test) for Cushion and Meditation conditions, and being reliably (p <.017, using Bonferroni correction) less than the Control group at T2 (except for VAS anxiety in the Cushion condition with p = .062).

The second interventions, at time T4 (post intervention 2), did not produce a significant difference in STAI anxiety between conditions as indicated by conducting between-subjects t-tests between Cushion and Control; t(87) = 1.44, p = .15, d = .305, and between Meditation and Control; t(82) = 1.364, p = .176, d = .298). Between time T3 (post test, pre intervention 2) and T4 (post intervention 2), all participants experience a significant reduction in anxiety levels with the removal of the anxiety-inducing stimulus.

**Fig 6:**
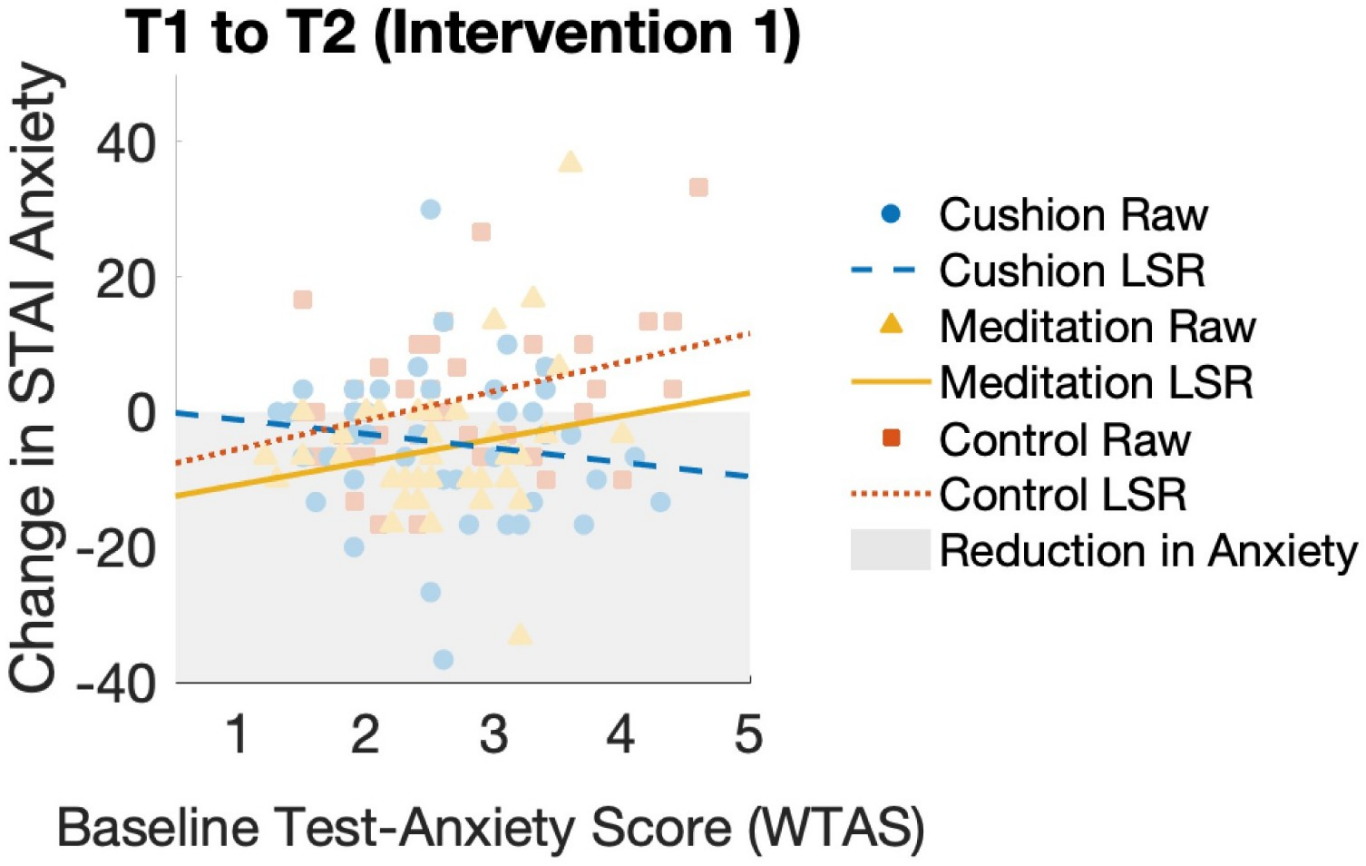
STAI anxiety scores against baseline Test-Anxiety scores between T1 and T2. Relationship between the participants’ change in STAI Anxiety scores and their baseline Test-Anxiety (WTAS) scores—from T1 (baseline) to T2 (post intervention 1, anticipating test). Line of best fit (LSR) shown for each condition using a linear Least Squares Regression model. Shaded regions highlight negative change in STAI, i.e. reduction in anxiety.

Conducting a mixed ANCOVA with Test Anxiety (WTAS) as the covariate measure, indicated a significant interaction between time and condition on STAI scores; F(3, 375) = 3.261, p = .025, $\eta_{p}^{2}=.025$. Inspecting the change in participant STAI anxiety scores between time T1 (baseline) and T2 (post intervention 1, anticipating test) in relation to participants baseline Test Anxiety (WTAS) scores indicates a difference between conditions as shown in Fig 6. A Pearson product-moment correlation was run to determine the relationship between participants’ trait Test-Anxiety (WTAS) and their change in STAI Anxiety between T1 (baseline) and T2 (post intervention 1, anticipating test). There was a medium, positive correlation for the Control group, which was statistically significant (r = .365, n = 44, p = .015). For the Cushion group, there was a small, negative correlation that was not statistically significant (r = -.151, n = 45, p = .323), and for the Meditation group there was a small, positive correlation that was not statistically significant (r = .204, n = 40, p = .207). In the case of the Cushion group, although the negative correlation is not statistically significant, it is significantly different to the correlation found for the Control group, using Fisher’s r to z transformation (z = 2.44, p = .0073). Although in the right direction, the Cushion group correlation coefficient was not significantly different to the Meditation group (z = 1.593, p = .056). The correlation coefficients for the Control and Meditation groups were not significantly different (z = 0.775, p = .219).

To enable a comparison between our intervention and that of Miri et al. [68], which is the only other large-scale study on the empirical effects of haptic breathing on anxiety reduction, we computed Cohen’s d for the reduction in STAI Anxiety of participants in the Cushion condition between time T1 (baseline) and T2 (post intervention 1, anticipating test). This resulted in an effect size of *d* = .41. Miri et al. [68] reported an effect size of *d* = .33 for the drop in STAI anxiety levels observed in their treatment group. Thus, both interventions represent a small to medium effect size. Note, for our intervention we would expect anxiety to actually be increasing between T1 (baseline) and T2 (post intervention, anticipating test), which was the case for the Control condition.

## 7 Discussion

In this study we have demonstrated through an empirical evaluation the anxiety-reducing effect of our final artefact—a breathing cushion interface—when students are anticipating an anxiety-inducing test. The design of this interface was informed by a qualitative evaluation of five initial prototypes with the target population of undergraduate students.

During the preliminary stage focus group, participants repeatedly found the breathing cushion prototype to have the most pleasant and calming interaction. There could be several contributing factors for this. Of the five prototypes, the breathing cushion was the ‘most natural’ since it was driven by hand (reducing mechanical sounds or vibrations from motors and inevitably adding some variability associated with living beings) and used pneumatics which more accurately replicates the mechanics of real breathing than, say, a vibration motor replicates purring.

In the empirical evaluation of the interface, we found evidence to suggest that holding the cushion interface reduces induced state anxiety in anticipation of a test compared to the control group and that the interface is as effective as a guided breathing meditation at reducing induced state anxiety. In support of the former, we found that STAI anxiety measures for participants in the cushion group were significantly lower than for those in the control group when anticipating the anxiety-inducing mathematics test. In support of the latter hypothesis, we found that the cushion intervention had a similar effect on STAI anxiety and self-reported VAS anxiety levels as the breathing meditation. This effect was noticed during the first intervention in anticipation of the test when STAI anxiety, VAS anxiety and VAS stress all decreased for the cushion and meditation conditions but not in the control condition.

However, the second intervention (post mathematics test) did not produce the same effect. At this time (T4, post intervention 2), there was no statistical difference in STAI anxiety between the conditions. It appears that the significant reduction in anxiety for all participants having completed the mathematics test nullified the previously recorded effect of the interventions. This indicates that both the cushion and meditation interventions may only be effective at reducing anticipatory test anxiety. However, a factor to take into consideration is the set-up of our experiment—in a real-world examination scenario, test-anxiety may continue post exam in the form of worry over exam results and the future implications of the student’s performance. In our experiment, there were no future implications of their performance in the test, so, once the test was completed, the cause of anxiety was removed.

When investigating the interaction between participants’ trait Test-Anxiety and their change in STAI anxiety during the anticipatory phase (T1, baseline to T2, post intervention 1), we found an interesting difference between the three conditions. In the control condition, the increase in STAI anxiety positively correlated with Test-Anxiety, as expected. However, in the cushion condition, a slight negative correlation was found that was significantly different to both the control and meditation conditions. This finding indicates that the cushion intervention may be particularly effective for students with higher trait Test-Anxiety. This finding was unique to the cushion and did not occur for the meditation condition, although a similar result was reported by Miri et al. [68] in their study with a vibrotactile breathing pacer. They found that participants with lower trait ‘cognitive reappraisal’ (based on the Emotion Regulation Questionnaire [95]) benefited more from the breathing pacer than those who scored higher on the scale. These findings indicate that haptic technologies may be particularly effective for people with lower trait capacity to regulate their emotions under stress.

During the intervention participants were only asked to hold the cushion interface and were not given any instructions, training or guidance. Compared to the meditation condition which provides continued guidance, there is no change or personalised interactivity in the cushion interface throughout the intervention, which could be expected to reduce its effectiveness. Since the cushion and meditation were found to have similar effect on anxiety, this provides encouraging evidence for the efficacy of the interface and that future iterations with more sophisticated behaviours could be yet more effective.

A further factor to consider is the duration of the interventions. The duration of 8 minutes 18 seconds was based on the meditation to ensure that all interventions were of equal duration. However, Cheng et al. demonstrate that the duration of deep breathing exercises has an effect on the activation of the parasympathetic nervous system [96]. They test four conditions, control (no breathing) and 5 minutes, 7 minutes and 9 minutes of deep breathing. They find that only the 9 minute duration is associated with the greater activation of the parasympathetic nervous system. Given this information, it could be that increasing the intervention duration has an increased effect in anxiety reduction, based on the assumption that the anxiety reduction is caused by the cushion encouraging deep breathing.

A consideration in the design of this kind of interface is that simulating human behaviour can be perceived as uncanny or creepy, negating the beneficial aspects of the cushion. All participants in the cushion condition (n = 45) gave feedback on their experience of the cushion and the vast majority of comments about the breathing aspect of the cushion were positive. Only two negative comments were made about the cushion breathing; one participant commented “I liked that the cushion was quite comforting but the fact it ‘breathed’ was a bit weird” and another said that they disliked that the breathing “felt quite artificial/robotic”. Other participants liked the interface but found the rate of breathing too deep/slow (4 participants) or hard to follow with their breath (3 participants) which could be amended in future iterations. One participant liked the interface and found it calming but found themselves getting bored over time. The lack of variation or responsiveness in the cushion could be the cause of this. However, the overwhelming majority of feedback was positive, with 33 participants describing the cushion as “calming”, “relaxing”, “comforting” or “pleasant”. The participant feedback indicates that, subjectively, the breathing cushion was primarily perceived positively, and was not associated with negative states such as awkwardness, creepiness or discomfort.

### 7.1 Limitations and future work

Many participants commented that the breathing cushion made them more aware of their breath or that they wanted to follow the rhythm of the cushion with their own breathing. We do not know if breath entrainment occurs when holding the cushion and we need to be cautious in considering what features of the breathing cushion cause the observed reduction in anxiety. Miri et al. [97] highlight some of the challenges in evaluating technologies for pacing breath. They report that their vibrotactile device successfully modulated breathing and reduced self-reported anxiety, but they did not find the expected reduction in skin conductance or that breathing parameters mediated self-reported anxiety. To identify the cause of anxiety reduction in the cushion will take careful further investigation. In particular, we note that this study did not compare the cushion with and without breathing; participants in the control and meditation conditions were not holding a static copy of the cushion. As such, the observed anxiety reduction could have been influenced by a novelty effect of the device. Additionally, the act of hugging the comforting cushion interface may be the primary contributing factor in reducing anxiety rather than the breathing behaviour. Sumioka et al. found that hugging a large human-shaped cushion reduced cortisol levels in participants when engaged in a phone call [49] and Zhang et al. found that hugging a pillow was most effective at relieving stress compared to other methods such as holding a stress ball, based on EEG data analysis [98]. Therefore, further experimentation is needed to isolate the effect of simulating breathing through the cushion.

Using biomarkers such as heart rate variability, breathing rate and skin conductance to monitor autonomic nervous system activity would provide useful evidence for the physiological effect of the interface as well as indicating whether breathing entrainment occurs when holding the cushion. Collecting this data would enable us to trace anxiety indicators in real-time during the onset and offset of anxiety-induction. In this study, the measures at time T4 (post intervention 2) were statistically indistinguishable between conditions, but this may have been due to the 8 minute duration between T3 (post test, pre intervention 2) and T4 (post intervention 2). The rate of anxiety reduction may not have been consistent across conditions during that interval and physiological data could shed light on this. It would also provide supporting data to mitigate the limitations of using self-report measures. For example, T3 (post test, pre intervention 2) measures were collected once participants had returned to their own rooms and this situation modification may have influenced their affective responses.

The minute ventilation of the cushion was based on a typical human adult. However, the shape and size of the cushion are not typical of a real human torso and the single inflatable chamber embedded in a plastic casing is a simplistic simulation of human lungs and ribcage. Future work will investigate in more detail what features of the cushion design—such as size, shape, stiffness, lung design and breathing characteristics—are key factors in providing anxiety relief and whether replicating human breath accurately is the most effective.

This study provides evidence that the breathing cushion interface should be developed further as an anxiety aid and investigated more thoroughly. The main focus of future work is to develop an untethered version of the interface and investigate the physiological effects of holding the cushion. The cushion form factor was designed to be huggable, but also to function as a normal cushion that could fit into the decor of a person’s home and be discreetly used as part of daily life. By developing an untethered interface we can test its efficacy in people’s daily home life and evaluate the practicalities of the interface longer term. In doing so we could learn how household members appropriate the cushion over time and capture their lived experiences, including when, why and where they decide to use it.

With an untethered prototype and a greater understanding of the physiological impact of the cushion, we can explore the efficacy of the interface for other populations identified as having high risk of experiencing anxiety. One of these populations is people with Dementia. People with Dementia have higher than averages rates of anxiety symptoms [99], which can be exacerbated when they have hospital appointments or situations that take them out of their familiar environment. A preliminary, exploratory focus group with three people with Dementia provided encouraging feedback that the interface may be accessible, beneficial and desirable to this population. All three participants expressed enjoyment when holding the cushion and wanted a version of it to take home.

## 8 Conclusion

In this study we have presented the design and testing of an intuitive haptic interface for easing state anxiety. A set of five primary prototypes were initially developed inspired by naturally occurring haptic sensations such breathing, heartbeat and purring. The prototypes were in the form of soft, huggable cushions to make them accessible and intuitive to interact with. Conducting a qualitative focus group indicated that the breathing cushion had demonstrably the most pleasant and calming behaviour of the five prototypes with no participants rating it as unpleasant. This led to the development of a larger version of the breathing cushion adapted to be mechanically driven, soft and ergonomic based on the focus group feedback. The breathing rate and shape of the interface were designed to encourage the slow, diaphragmatic breathing associated with easing anxiety.

This interface was formally tested in a psychometric experiment with 129 participants, comparing it to breathing meditation and a control case. Baseline anxiety, resilience, mindfulness and stress measures were statistically similar across condition groups. When anticipating an anxiety-inducing event (a verbal mathematics test in front of people), participants in the Cushion and Meditation conditions had a significantly reduced level of anxiety (measured by STAI) compared to participants in the control condition. The experiment demonstrated that holding the breathing cushion interface is an effective alternative to mindful breathing practices at reducing anxiety without need for training or guidance.

In conclusion, the breathing cushion interface is an intuitive haptic device shown to ease state anxiety in the student population when anticipating a test. This suggests that the interface could be an alternative non-pharmaceutical anxiety aid for students during examination periods. The development of an untethered model that can be trialled in people’s homes is our main future goal.

## Supporting information

S1 Fig Participant descriptions of the primary prototypes. Word clouds of adjectives or emotive phrases participants used to describe the 5 different prototypes during the focus group. Common phrases are assigned to a theme, for example ‘positive association’ refers to participants saying phrases such as ‘I like the cushion it reminds me of my pet’. Frequency of each word/phrase shown in brackets.
(TIF)

S1 Video Prototype mechanisms and behaviours. This video shows and discusses the internal mechanisms of the prototypes and the final artifact in more detail and demonstrates them in action.
(MP4)
