# Supplementary figures and images for: A calming hug: Design and validation of a tactile aid to ease anxiety
Source: PLoS One. 2022 Mar 9;17(3):e0259838. doi: 10.1371/journal.pone.0259838 (PMC8906645; doi:10.1371/journal.pone.0259838)

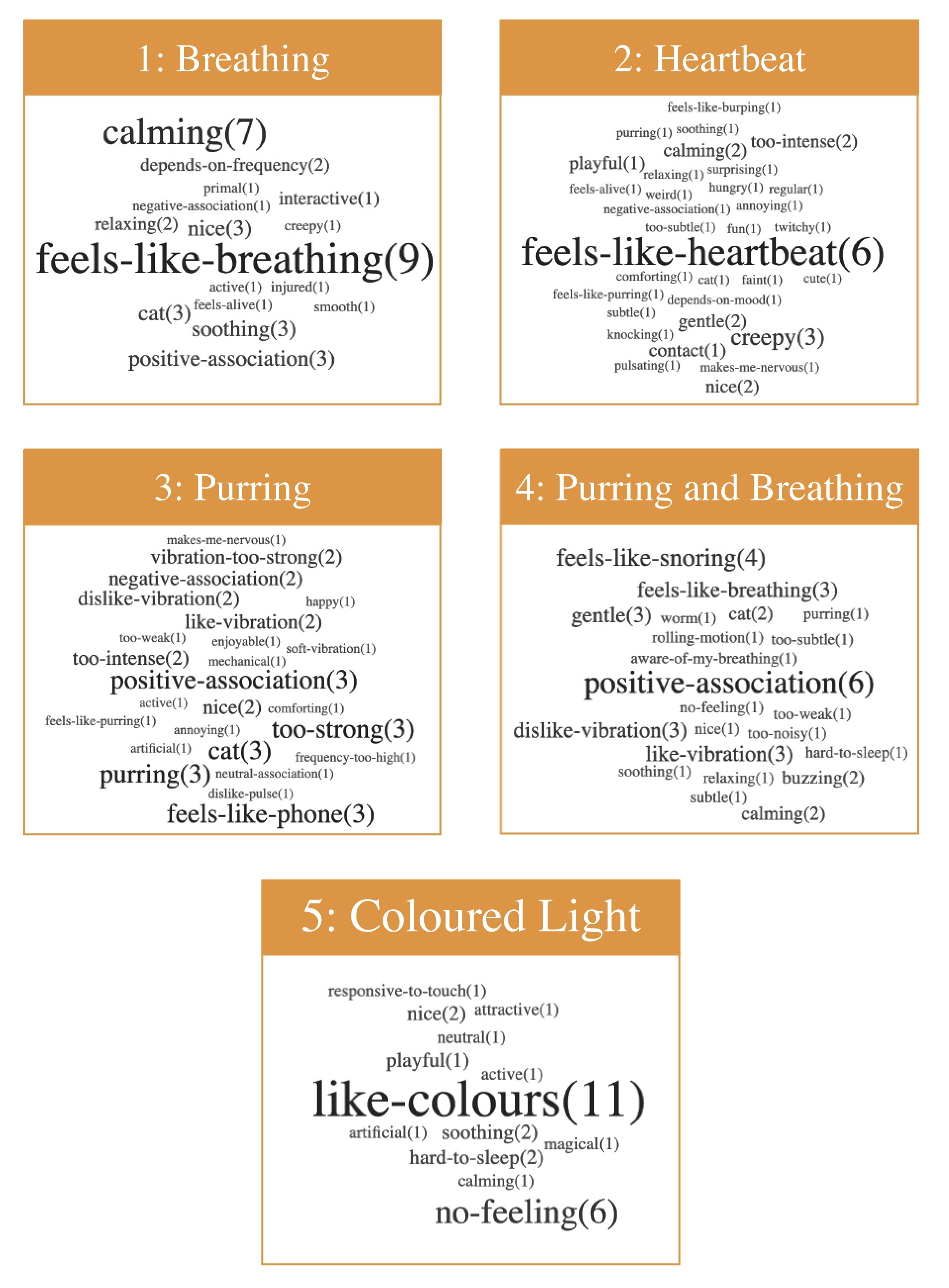

Supplement: S1 Fig — Word clouds of adjectives or emotive phrases participants used to describe the 5 different prototypes during the focus group. Common phrases are assigned to a theme, for example ‘positive association’ refers to participants saying phrases such as ‘I like the cushion it reminds me of my pet’. Frequency of each word/phrase shown in brackets. (TIF) [file pone.0259838.s001.tif]
